# Plasma volume expansion and capillary leakage of 20% albumin in burned patients and volunteers

**DOI:** 10.1186/s13054-020-02855-0

**Published:** 2020-05-05

**Authors:** Markus Zdolsek, Robert G. Hahn, Folke Sjöberg, Joachim H. Zdolsek

**Affiliations:** 1 Department of Biomedical and Clinical Sciences (BKV), Linköping University, Linköping, Sweden; 2 Research Unit, Södertälje Hospital, Södertälje, Sweden; 3 Department of Clinical Sciences at Danderyd Hospital (KIDS), Karolinska Institutet, Stockholm, Sweden

**Keywords:** Burns (physiology), Capillary permeability (physiology), Serum albumin (pharmacokinetics, therapy)

## Abstract

**Background:**

Burn injury is associated with a long-standing inflammatory reaction. The use of albumin solutions for plasma volume support is controversial because of concerns of increased capillary leakage, which could aggravate the commonly seen interstitial oedema.

**Methods:**

In the present open controlled clinical trial, an intravenous infusion of 20% albumin at 3 mL/kg was given over 30 min to 15 burn patients and 15 healthy volunteers. Blood samples and urine were collected for 5 h. Plasma dilution, plasma albumin and colloid osmotic pressure were compared. Mass balance calculations were used to estimate plasma volume expansion and capillary leakage of fluid and albumin.

**Results:**

The patients were studied between 4 and 14 (median, 7) days after the burn injury, which spread over 7–48% (median, 15%) of the total body surface area. The albumin solution expanded the plasma volume by almost 15%, equivalent to twice the infused volume, in both groups. The urinary excretion exceeded the infused volume by a factor of 2.5. Capillary leakage of albumin occurred at a rate of 3.4 ± 1.5 g/h in burn patients and 3.7 ± 1.6 g/h in the volunteers (*P* = 0.61), which corresponded to 2.4 ± 1.0% and 2.5 ± 1.2% per hour of the intravascular pool (*P* = 0.85). The median half-life of the plasma volume expansion was 5.9 (25th–75th percentiles 2.7–11.7) h in the burn patients and 6.9 (3.4–8.5) h in the volunteers (*P* = 0.56).

**Conclusions:**

Albumin 20% was an effective volume expander in patients at 1 week post-burn. No relevant differences were found between burn patients and healthy volunteers.

**Trial registration:**

EudraCT 2016-000996-26 on May 31, 2016.

## Introduction

Albumin is the colloid fluid of choice when treating oedematous hypovolemic patients. A comparison between iso-oncotic 4–5% and the hyperoncotic 20% albumin in intensive care patients showed a fluid-sparing effect of the latter preparation [1], which is desirable because oedema impairs wound healing [2] and increases the diffusion distance for oxygen [3].

Burn patients develop severe oedema due to negative interstitial fluid pressure [4], inflammation-induced tissue swelling and intensive crystalloid fluid therapy. The use of albumin is controversial. A widespread belief is that infused albumin is easily translocated to the interstitial space because the injury elicits an inflammation-mediated toxic effect on the endothelium [5–9]. However, the intravascular persistence of 20% albumin and the associated capillary leakage has rarely been assessed in a clinical setting.

The aim of this study was to compare the plasma volume expansion in burn patients with that of volunteers following administration of a standardised amount of 20% albumin (primary outcome measure). A further purpose was to assess the changes in colloid osmotic pressure (secondary outcome measure) and to use these data to estimate the capillary leakage of albumin and fluid.

The hypothesis was that burn patients have a less pronounced plasma volume expansion and a lower colloid osmotic pressure, but a greater capillary leakage of albumin and fluid. For ethical reasons, patients were not studied during the most active resuscitation stage, but instead between 4 and 14 days (mean, 7 days) after the burn injury. The assessments were made using mass balance calculations based on measurements of blood Hb, plasma albumin and urine.

## Methods

This study was an open-label interventional controlled clinical trial that compared the volume effects of albumin in 15 burned patients with those in 15 healthy volunteers.

Patients were recruited at the National Burn Intensive Care Unit at Linköping University Hospital during a visit 1–2 days before the trial. All participants gave their informed consent orally and in writing. Inclusion criteria were a burn area > 6% of the total body surface area (TBSA) and age between 18 and 80 years. Exclusion criteria were unconscious patients or those with severe allergies, kidney failure or heart failure.

The study was performed on 14 patients in the morning and on 1 patient in the evening. All patients were haemodynamically stable and had fasted overnight, but were allowed to ingest one sandwich and drink one glass (200 mL) of liquid 1.5 h prior to the experiment. Some patients were treated with intravenous antibiotics and could have received 100 mL of 0.9% saline as a vehicle during the fasting period.

The patients were placed in the supine position for at least 30 min before obtaining the baseline measurements. All patients emptied their bladders prior to the start of the study, either voluntarily by voiding or via a urinary catheter. Patients received an infusion of 3 ml/kg of 20% albumin over 30 min. Blood samples were drawn at 15 occasions during the 5 h following the start of the infusion, using either a venous cannula or a pre-existing radial artery cannula. Baseline samples were drawn in duplicate, and the mean values were used in calculations. A small volume of blood was drawn from the cannula and re-administered, together with 2 mL of 0.9% saline, to avoid sample dilution. Urine samples were collected from a catheter bag or through voluntarily voiding at 0 and 5 h. Patients were not allowed to eat, sit up or raise their legs during the 5 h of the study.

The 15 healthy volunteers (9 males and 6 females), who did not take daily medication, were recruited through posters at the hospital where the study was performed. Exclusion criteria were pregnancy and severe allergy. These control patients underwent the same pre-study fasting, albumin infusion and follow-up as the burn patients. Data from this control group has previously been published [10, 11].

### Blood and urine chemistry

Analyses of blood samples were conducted in the hospital’s central laboratory. The blood haemoglobin (Hb) concentration was sampled in EDTA tubes and measured on a Cell-Dyn Sapphire (Abbott Diagnostics, Abbott Park, IL). The coefficient of variation (CV) for this analysis was 1.2%, as ensured by the duplicate samples taken at baseline.

The blood was also sampled in lithium-heparin plasma gel tubes for the measurement of the concentrations of creatinine, albumin, C-reactive protein (CRP) and interleukin-6 (IL-6), and the urine osmolality was measured on a Cobas® 8000 (Roche Diagnostics, Basel, Schweiz) with CVs of 2%, 3.2% (duplicates), 1.9–4.2%, 2.5% and 3.0%, respectively.

The colloid osmotic pressure (COP) at all 15 sampling points was measured in duplicate in our research laboratory on an Osmomat 050 (Gonotec, Berlin) with a CV of 1.9% within 1–3 days after the experiment’s completion.

### Calculations

The initial blood volume was obtained using Nadler’s formula, where the total blood volume prior to the infusion of 20% albumin (BV_0_) was derived from the height (*h*) in metres and weight (*w*) in kilogrammes and expressed according to gender [12].

The total mass of haemoglobin at the baseline (MHb_0_) was estimated using a mass balance equation based on the BV_0_ and the Hb concentration at the baseline (Hb_0_):

$$ {\mathrm{MHb}}_0={\mathrm{BV}}_0\ \mathrm{x}\ {\mathrm{Hb}}_0 $$

The loss of Hb caused by blood sampling at time (*t*) was subtracted from MHb_0_. The BV(*t*) at a later time (*t*) was obtained by division with a new measurement of Hb [10]:

$$ {\mathrm{MHb}}_t={\mathrm{MHb}}_0-{\mathrm{MHb}}_{\mathrm{loss}\ t} $$

$$ {\mathrm{BV}}_t=\frac{{\mathrm{MHb}}_t}{{\mathrm{Hb}}_t} $$

Plasma volume (PV) was obtained as

$$ {\mathrm{BV}}_t\ \left(1-{\mathrm{Hct}}_t\right)={\mathrm{PV}}_t $$

The capillary leakage of albumin was obtained as the change in albumin mass with correction for the infused amount of albumin [11]:

$$ \mathrm{Albumin}\ \mathrm{leak}=\mathrm{Infused}\ \mathrm{albumin}+\left({\mathrm{PV}}_{\mathrm{o}}\ P-{\mathrm{Alb}}_{\mathrm{o}}\right)-\left({\mathrm{PV}}_t\ P-{\mathrm{Alb}}_t\right) $$

The half-life of the infused albumin was calculated from the decrease in the product of *P*-alb and the Hb-derived plasma dilution over time.

### Statistics

The study was powered to detect a difference of 5%, at a certainty of 80% and a significance level of *P* < 0.05, for the plasma volume expansion at the end of the infusion. The effect size was 1.04, as reported previously [11].

The results are reported as mean ± standard deviation. Differences between the groups were evaluated by one-way or repeated measures analysis of variance. Data with a skewed distribution were reported as the median (25th–75th percentiles). The groups were compared using the Mann-Whitney *U* test, and changes over time were evaluated by Friedman’s test. Correlations between variables were studied by simple linear regression, where *r* = correlation coefficient. *P* < 0.05 was considered statistically significant.

## Results

The burn patients were studied between October 2016 and January 2019 and the healthy volunteers between October and December 2016. There were no patient or healthy participant exclusions. The demographic details and basic data on the experiments are shown in Table 1.

**Table 1:** Demographics and basic measurements

Variable	Hour	Burn patients	Volunteers	Statistics
Demographics				
Subjects (*N*)		15	15	
Females/males (*N)*		3/12	6 / 9	*P* = 0.23
Age (years)		45 ± 15	31 ± 12	*P* < 0.010
Body weight (kg)		95 ± 17	76 ± 13	*P* < 0.003
Body mass index (kg/m^2^)		29.3 ± 5.2	25.2 ± 4.1	*P* < 0.03
Measurements				
Mean arterial pressure (mmHg)	0	87 ± 10	93 ± 6	*P* = 0.074
	1	87 ± 8	91 ± 6	*P* = 0.13
	5	88 ± 9	90 ± 7	*P* = 0.50
Plasma IL-6 concentration (ng/L)	0	38 (20–91)	1.7 (1.5–2.2)	*P* < 0.001
	1	37 (19–60)	1.9 (1.5–2.6)	*P* < 0.001
	5	48 (18–71)	3.4 (2.3–4.3)	*P* < 0.001
Plasma C-reactive protein (μg/L)	0	86 (50–155)	1.0 (0.3–5.0)	*P* < 0.001
	1	69 (44–132)	0.5 (0.3–1.5)	*P* < 0.001
	5	72 (42–140)	0.4 (0.2–2.0)	*P* < 0.001
Plasma albumin (g/L)	0	24.3 ± 4.7	38.3 ± 2.7	*P* < 0.001
	1	31.8 ± 4.5	44.7 ± 2.5	*P* < 0.001
Plasma creatinine (μmol/L) *	0	78 ± 13	78 ± 20*	*P* = 0.99
	5	77 ± 10	72 ± 19	*P* = 0.40
Urine osmolality (mosmol/kg)	0	657 (541–740)	774 (433–855)	*P* = 0.40
	5	563 (481–683)	419 (336–673)	*P* = 0.32
Urine creatinine (mmol/L)	0	11.5 (7.9–14.0)	16.0 (8.1–26.3)	*P* = 0.13
	5	8.8 (5.8–11.0)	7.8 (4.2–14.3)	*P* = 0.83

*Data missing on 2 volunteers

The patients were studied between 4 and 14 (median, 7) days after their burn injuries, which covered between 7 and 48% (median, 15%) of the TBSA.

### Measurements

**Fig. 1:**
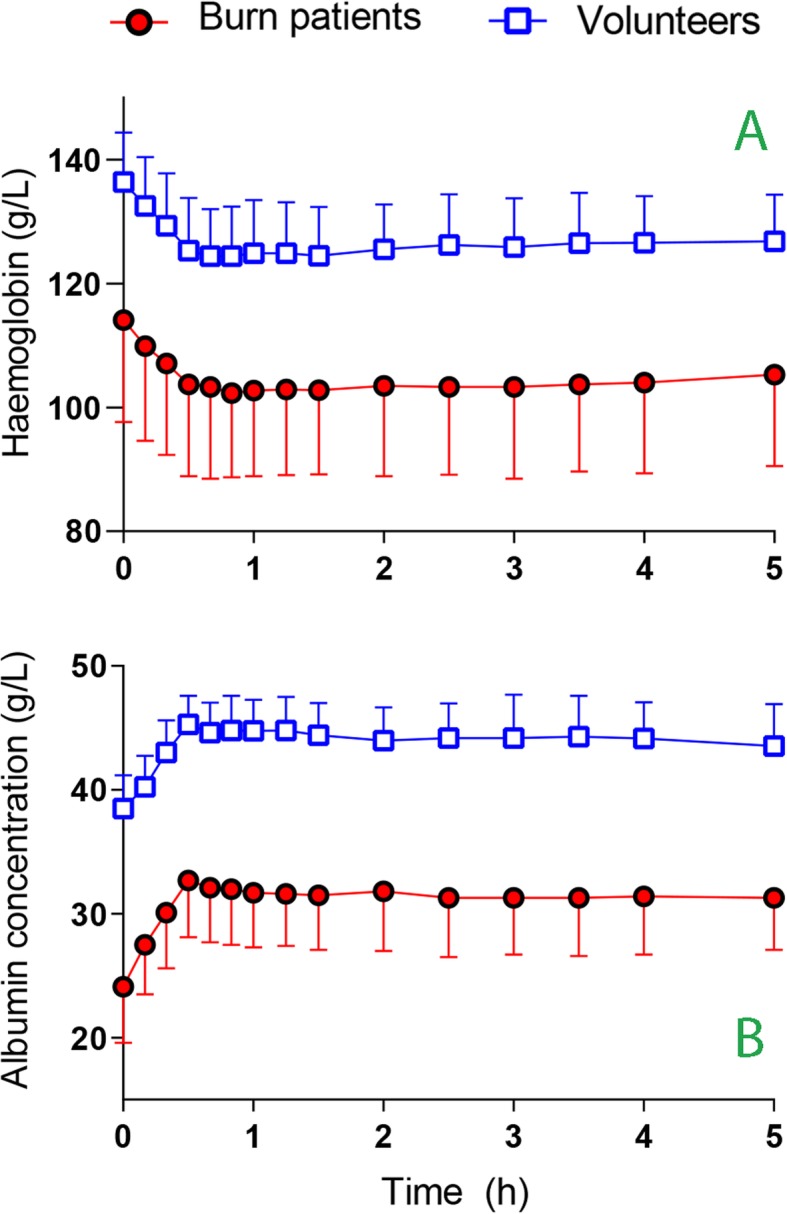
**a** The blood haemoglobin and **b** plasma albumin concentration during and after infusion of 3 ml/kg of 20% albumin over 30 min in burn patients and volunteers. Data are the mean (SD)

**Fig. 2:**
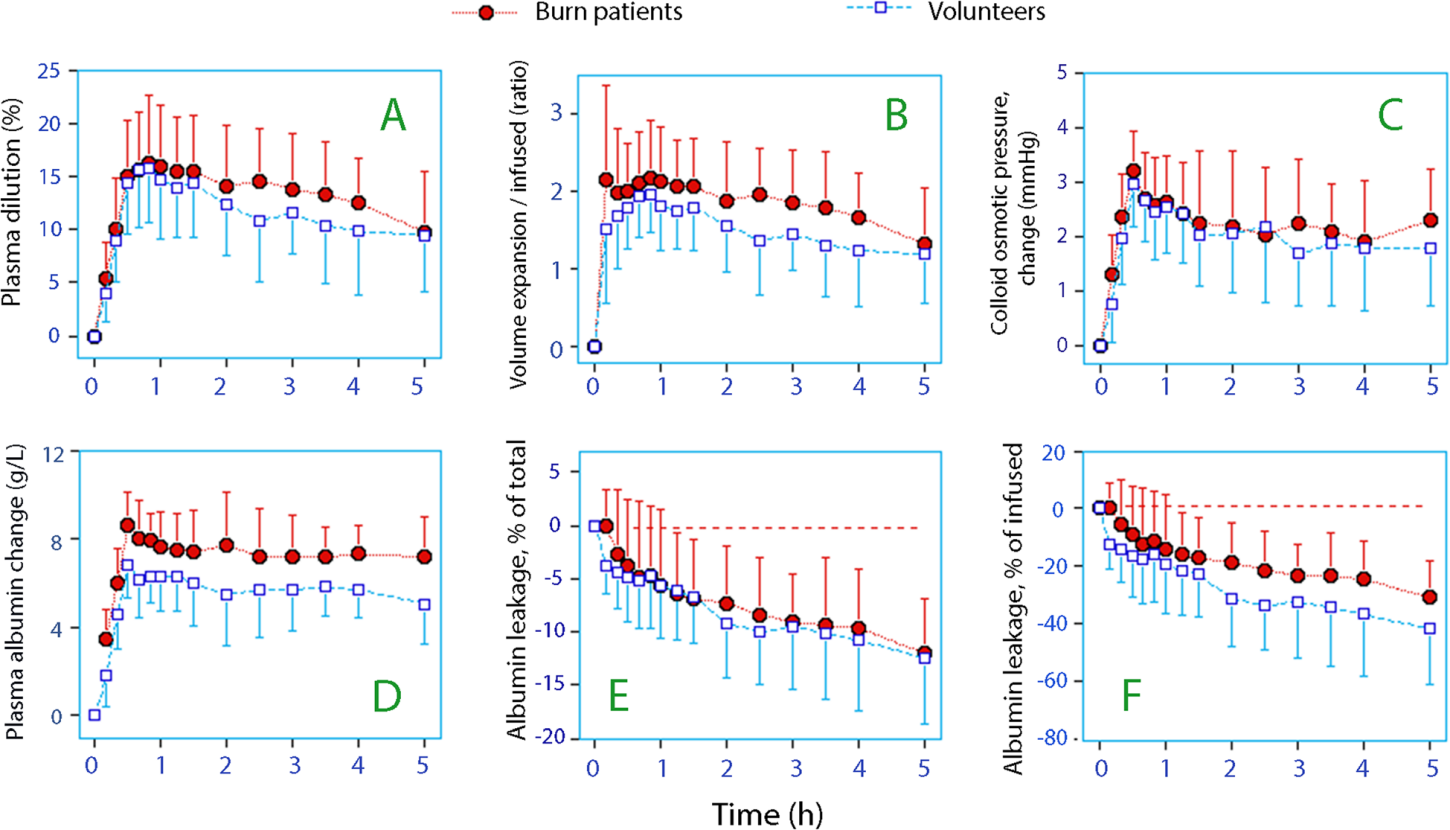
**a** Plasma dilution. **b** Plasma volume expansion divided by the infused volume of 20% albumin. **c** Changes in plasma oncotic pressure. **d** Changes in plasma albumin concentration. **e** Capillary leakage of albumin expressed as a percentage of the total amount of albumin in the plasma and **f** expressed as a percentage of the infused amount of albumin. All data are the mean (SD)

Figure 1 shows the crude blood Hb and plasma albumin concentrations. The greatest plasma dilution, based Hb changes, occurred 20 min after the infusions ended; this dilution was 16.3 ± 6.0% in the burn patients and 15.8 ± 4.9% in the volunteers (*P* = 0.80, Fig. 2a).

The estimated increase in plasma volume amounted to twice the infused fluid volume (Fig. 2b, Table 2). The total recruitment of fluid from the extravascular space over the 5 h of the study was 3.2 ± 1.8 mL for every millilitre of infused 20% albumin in the patients and 3.4 ± 1.1 mL in the volunteers (*P* = 0.75).

**Table 2:** Fluid volume calculations

Variable	Hour	Burn patients	Volunteers	Statistics
Blood volume (L)	0	5.7 ± 0.8	4.9 ± 0.9	*P* < 0.008
Plasma volume (L)	0	3.8 ± 0.7	2.9 ± 0.5	*P* < 0.004
Infused fluid volume (mL)	0.5	282 ± 56	229 ± 39	*P* < 0.006
Plasma volume expansion (L)	1	0.59 ± 0.22	0.43 ± 0.18	*P* < 0.03
	0–5	0.50 ± 0.18	0.35 ± 0.14	*P* < 0.02
Plasma volume expansion/infused (ratio)	1	2.2 ± 0.7	1.9 ± 0.5	*P* = 0.28
	0–5	1.8 ± 0.5	1.5 ± 0.4	*P* = 0.12
Intravascular half-life (h)				
Fluid	0–5	5.9 (2.7–11.7)	6.9 (3.4–8.5)	*P* = 0.56
Albumin	0–5	10.3 (8.0–16.1)	6.0 (4.3–7.2)	*P* < 0.004
Missing fluid/albumin		6 / 3	3 / 2	
Creatinine clearance (mL/min)	0–5	196 (156–242)	186 (158–205)	P = 0.81
Fractional sodium excretion (%)	0–5	0.83 (0.67–1.18)	0.43 (0.17–0.88)	P = 0.15

The COP prior to the infusion was 19.2 ± 2.6 mmHg in the burn patients and 26.4 ± 1.8 mmHg in the volunteers (*P* < 0.001). The maximum increase in COP was 3.2 ± 0.7 mmHg in the burn patients and 3.0 ± 0.8 mmHg in the volunteers at the end of the infusions (*P* = 0.40). The COP changes over time were quite similar (*P* = 0.50, Fig. 2c).

The plasma albumin concentration increased in both groups but was lower overall in the patients (Fig. 2d, Table 1). The mean increase in plasma albumin over the 5-h study period was greater in the burn patients, at 7.1 ± 1.4, versus 5.6 ± 1.2 g/L for the volunteers (*P* < 0.001).

The plasma concentrations of IL-6 and CRP were considerably higher in the burn patients than in the volunteers (Table 1). In both groups, IL-6 levels increased during the study (*P* < 0.001, Friedman’s test), whereas CRP levels did not change significantly.

The urinary excretion was 86 (79–198) mL/h in the burn patients and 100 (73–155) mL/h in the volunteers during the 5 h of the study (*P* = 0.80). The excreted urine was 2.5 ± 1.5 times greater than the infused volume of 20% albumin in the patients and 2.7 ± 1.5 times greater in the volunteers (*P* = 0.64).

### Calculations

Capillary leakage of albumin occurred at a rate of 3.4 ± 1.5 g/h in the burn patients compared to 3.7 ± 1.6 g/h in the healthy volunteers (*P* = 0.61).

This leakage of albumin corresponded to 2.4 ± 1.0% of the intravascular pool per hour in the burn patients and 2.5 ± 1.2% per hour in the volunteers (*P* = 0.85, Fig. 2e). When expressed as a percentage of the administered amount, the leakage of albumin at 5 h tended to be smaller in the burn patients, as it represented 6.2 ± 2.4% per hour in the burn patients compared to 8.4 ± 3.7% per hour in the volunteers (*P* = 0.08, Fig. 2f).

The median half-life of the plasma volume expansion was 5.9 h (25th–75th percentiles 2.7–11.7) in the burn patients and 6.9 (3.4–8.5) in the volunteers (*P* = 0.56). The intravascular persistence of the infused albumin had a half-life of 10.3 (8.0–16.1) h in the burn patients and 6.0 (4.3–7.2) h in the volunteers (*P* < 0.004). However, the half-lives of 14 of the 60 elimination curves could not be estimated due to prolonged steady state (Table 2).

**Fig. 3:**
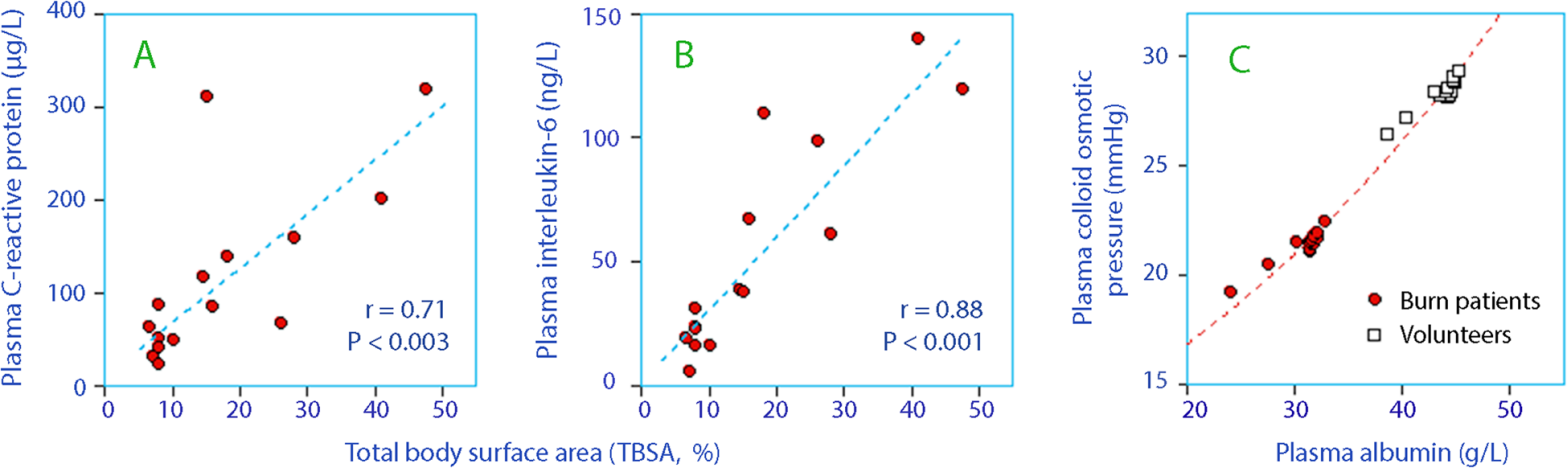
Linear relationship between the burned area (as the percentage of total body surface area, TBSA) and **a** the C-reactive protein concentration in plasma, and **b** the interleukin-6 concentration in plasma. Each point is one patient in the burned group. **c** Logarithmic correlation between plasma albumin and the plasma colloid osmotic pressure. Each point is the mean values of 15 subjects. Frequent overlapping

A statistically significant relationship was found between the burned body surface area and the baseline plasma concentrations of CRP (Fig. 3a) and IL-6 (Fig. 3b). Plasma albumin correlated with COP in a logarithmic fashion (Fig. 3c). No statistically significant relationship was detected between the inflammatory biomarkers and the time lapse between the burn injury and the experiment.

A post hoc estimation of the mean baseline plasma volume in both groups was made based on the plasma dilution and plasma albumin concentration at 40 min (= 2/3 h), using a correction for the calculated half-life (*T*_1/2_) of the infused albumin:

$$ \mathrm{P}-{\mathrm{alb}}_t\ {\mathrm{PV}}_{\mathrm{o}}\ \left(1+{\mathrm{dilution}}_t\right)=\mathrm{P}-{\mathrm{alb}}_{\mathrm{o}}\ {\mathrm{PV}}_{\mathrm{o}}+\mathrm{infused}\ \mathrm{albumin}\ \left[e\hat{\mkern6mu} \left(\left(-\ln\ 2/{T}_{1/2}\right)\ 2/3\right)\right] $$

The estimated PV_o_ was multiplied with the *F* cell ratio of 0.91 to obtain the same format for albumin distribution as Nadler et al. [12]. PV_o_ was then 3.76 L in the burned patients and 2.96 L in the volunteers, which should be compared to the 3.8 and 2.9 L obtained with Nadler’s regression equations.

## Discussion

The plasma volume expansion in response to 20% albumin was quite similar in the burn patients and healthy volunteers, despite the fact that the burns had developed an inflammatory response to the injury. Plasma albumin and COP showed different values at baseline, but the groups followed each other closely during the treatment with 20% albumin. The capillary leakage of fluid and albumin was quite similar between the groups but, due to higher body weight in the burned patients, the leakage tended to be smaller in them when expressed in per cent of the infused amount.

Treatment with albumin in burns has previously been controversial, in particular when given early after the injury [13–16], although albumin might reduce the mortality [17]. The present results show that 20% albumin is an effective plasma volume expander with a prolonged intravascular persistence. Capillary leakage of albumin was coupled with a reduction of the plasma volume expansion, which sometimes left the plasma albumin and COP at a steady state throughout the 5 h of the study [11, 18]. The half-life of the induced plasma volume expansion averaged 6–7 h while the albumin molecules remained in the plasma with a half-life for between 6 and 10 h. However, in 23% of the washout curves, the elimination was so slow that the half-life could not be determined with confidence, indicating that the study period should have been longer than 5 h to obtain reliable half-lives in all subjects.

The use of hyperoncotic albumin is motivated by a presumed strong plasma volume expansion by virtue of recruitment of fluid from non-circulating sources, presumably from the interstitial fluid. However, a concern has been that inflammatory reactions would accelerate the capillary leakage of albumin [5, 7, 14] probably by causing degradation of the endothelial glycocalyx layer [8, 9]. The present study shows that the long-standing inflammation following burn injury does not alter the kinetic profile of 20% albumin when given approximately 1 week after the injury. The hyperoncotic albumin preparation also has a quite stable kinetic profile after major surgery, as the patients show plasma volume expansion and capillary leakage almost identical to that of volunteers [11]. Exogenous albumin is believed to have a protective effect on the glycocalyx due to its ready binding to the glycocalyx surface [19]. Therefore, the current finding should not be uncritically extrapolated to other fluids.

Biomarkers of inflammation were increased in the burn patients, and the severity varied with the burned area. These observations agree with previous work [20, 21] and emphasise the inflammatory potential of burn injuries. At the average time point of the study (7 days), the wounds are colonised by bacteria, which would further aggravate the inflammatory response. Therefore, no relationship was noted between the inflammatory intensity and the time lapse that occurred between the injury and initiation of our study.

The urine flow rate is expected to decrease when plasma albumin is rapidly increased. The reason is that a high intravascular COP counteracts glomerular filtration. On the other hand, fluid-induced plasma volume expansion increases the glomerular filtration, probably by improving perfusion of the kidneys [22]. The latter was apparently the stronger physiological stimulus in the present study, as the albumin infusions increased the urine flow rate. Overall, the subjects voided 2.5 times more fluid than the volume they received. The accelerated urine flow is demonstrated by the decrease in urine osmolality and urinary creatinine concentration during the experiment. Assuming a constant excretion of creatinine over time, the urine flow rate increased by 35% in the burned patients and by 97% in the volunteers. The urinary creatinine concentration was 40% higher in the volunteers than in the burn patients before the infusions started [23], which confirms that dehydration was not an issue in the latter group. Moreover, patients with such concentrated urine are known to show a greater increase in glomerular filtration rate on plasma volume expansion than other patients do [22], which explains the greater increase in urinary excretion among the volunteers.

Crystalloids are the most widely used resuscitation fluid in burn injuries [15, 16]. These fluids readily distribute across the capillary wall and cause interstitial oedema, particularly when infused at a high rate [24]. Early in the course of a burn injury, crystalloid-induced oedema is worsened by an aggravation of the negative interstitial pressure, which is probably a consequence of the acute degradation of collagen [25, 26]. The phase of strongly negative interstitial pressure should have been over when the present study was performed, but the burn patients were still in a long-standing inflammatory state in which the endothelial glycocalyx layer was unlikely to be intact.

### Limitations

The experiments described here were carried out in a strictly standardised fashion, but the body weights and the plasma concentrations of certain key variables differed between the groups at baseline. Heavier subjects would have received more 20% albumin, so the plasma dilution and the plasma volume expansion differed in numerical (Table 2), although not in relative, terms (Fig. 2a, b). Therefore, the result of our evaluation of the primary outcome measure is that the plasma volume expands in proportion to the infused volume of 20%, with no relevant differences between burn patients and healthy volunteers.

The secondary outcome measures were derived from plasma albumin, COP, and body weight, which all differed at baseline. The albumin infusions gave greater rises in plasma albumin in the burn patients, which can be understood from their hypoalbuminaemic state (Fig. 2d). The albumin leakage was similar in both groups in absolute, but not in relative, terms, suggesting that capillary leakage of albumin was relatively independent of the albumin concentration in the plasma (Fig. 2e and f). The burn patients also had a markedly lower COP at baseline; however, absolute changes between the groups in response to the infusions were apparently cancelled out due to the logarithmic correlation between the plasma albumin and COP [27].

A further limitation is that ethical considerations precluded the inclusion of patients with the very largest burn injuries. The burned area of our patients varied between 7% and 48% of the TBSA, which is still representative of the clinical scenario for burns at an intensive care unit. In addition, the most acute stage (days 1 to 3) was not covered in the present study, which was due to ethical and practical problems associated with the extensive study protocol. The fluid that evaporated from the burned area was also not included in the calculations.

A minor but statistically significant increase was noted in the plasma IL-6 concentration in the burn victims during the study. This increase might be due to inflammation caused by the blood sampling procedure, where some withdrawn blood was re-administered to keep the sampled blood volume as low as possible (mean total 150 mL).

Mass balance has the benefit of being based on fewer assumptions when compared with radioactive tracer methods. The calculations made to create Fig. 2e and f assume an even distribution of the infused fluid and albumin in the vascular compartment. Figure 2b and the plasma volume correction for blood sampling also assume that the baseline plasma volume can be correctly estimated by anthropometric methods. However, in the present study, we confirmed the estimated baseline plasma volume in the two groups by a post hoc analysis of data.

## Conclusions

Infusion of 20% albumin induced a long-lasting plasma volume expansion that amounted to twice the infused volume, as well as a urinary excretion that was 2.5 times the infused volume. The effectiveness was similar in patients studied at an average of 7 days post-burn when compared with healthy volunteers, despite marked differences in inflammatory biomarkers.

## Data Availability

The original data can be obtained from the corresponding author.

## Abbreviations

BV: Blood volume
CRP: C-reactive protein
COP: Colloid osmotic pressure
CV: Coefficient of variation
Hb: Haemoglobin
IL-6: Interleukin-6
MHb: Haemoglobin mass
TBSA: Total body surface area
